# A Model of Judicious Advertising

**Published:** 1911-09-30

**Authors:** Hy. J. Eason


*To the Editor of* The Hospital.

### "A MODEL OF JUDICIOUS ADVERTISING."

*To the Editor of* The Hospital.

Sir,—In the issue of The Hospital of September 23 I observe that a mistake has arisen with regard to the pamphlet recently published in the interests of the Manchester Children's Hospital.

The pamphlet in question was written by the Chairman of the Board of Governors with a view to securing increased support for the work of the charity.

I had the pleasure and the privilege of being associated with the Chairman in its compilation, but the idea originated with him, and it is to Mr. Heywood our thanks are due for this interesting departure from the usual stereotyped form of appeal.—Yours faithfully, Hy. J. Eason.

September 26.

